# The Parametric, Psychological, Neuropsychological, and Neuroanatomical Properties of Self and World Evaluation

**DOI:** 10.1371/journal.pone.0031509

**Published:** 2012-02-13

**Authors:** Alan N. Simmons, Rachel E. Thayer, Andrea D. Spadoni, Scott C. Matthews, Irina A. Strigo, Susan F. Tapert

**Affiliations:** 1 Mental Health, VA San Diego Healthcare System, San Diego, California, United States of America; 2 Department of Psychiatry, University of California San Diego, San Diego, California, United States of America; RAND Corporation, United States of America

## Abstract

**Background:**

As an individual moves from adolescence to adulthood, they need to form a new sense of self as their environment changes from a limited to a more expansive structure. During this critical stage in development the last dramatic steps of neural development occur and numerous psychiatric conditions begin to manifest. Currently, there is no measure that aids in the quantification of how the individual is adapting to, and conceptualizing their role in, these new structures. To fill this gap we created the Self and World Evaluation Expressions Test(SWEET).

**Method:**

Sixty-five young adults (20.6 years-old), 36 with a history of drug use, completed the SWEET. A factor analysis was performed on the SWEET and the resultant factors were correlated with psychological, neuropsychological, and neuroanatomical battery that included both T1-wieghted and diffusion tensor magnetic resonance imaging scans.

**Results:**

We derived four factors: Self, Social-Emotional, Financial-Intellectual, and Spirituality. While showing limited relationships to psychological and neuropsychological measures, both white matter integrity and gray matter density showed significant relationships with SWEET factors.

**Conclusions:**

These findings suggest that while individual responses may not be indicative of psychological or cognitive processes they may relate to changes in brain structure. Several of these structures, such as the negative correlation of the affective impact of world with the dorsal anterior corpus callosum white matter integrity have been observed in psychiatric conditions (e.g., obsessive-compulsive disorder). Further longitudinal research using the SWEET may help understand the impact of dramatic shifts in self/world conceptualization and potentially link these shifts to underlying changes in brain structure.

## Introduction

Having a realistic conceptualization of one's influence on the world, as well as the impact of the external world on oneself is necessary for adaptive functioning [1]. Gaining such a conceptualization may occur during individuation, and be one of the final stages in the development of self, occurring at the point when an individual begins to consider the implications of life outside of the direct influence of parents and other entities that provide structure during childhood and adolescence [2]. Children believe that their relative importance within the world is substantial, which may be due to the fact that children usually live within smaller and more confined social environments (i.e., within their family structure and in the classroom). By comparison, the social environments that adults usually live in are larger and more complex [3], and adults usually realize that a single individual is less likely to have a substantial impact on large societal structures. As our culture becomes more urban and globalized, not only do these structures expand, but the number of individuals who are faced with these sudden shifts from small to vast surrounding structures increases [4]. An individual's conceptualizations about their impact on the world, and the impact of the world on them, are likely related to the individual's emotional, social, intellectual, financial, and spiritual functioning. However, prior research has not examined the emotional, social, intellectual, financial, and spiritual domains of self and world impact evaluation in adults and adolescents, and there have been no neuroimaging studies in this area.

Research on brain changes throughout the lifespan has focused on the frontal lobes as one of the last regions to develop completely [5], [6], [7]. Specifically, frontal lobe white matter volumes typically increase from childhood to early adulthood [8], which is likely due to increased myelination [9], [10], [11]. In contrast, gray matter volumes tend to decrease [5] because of synaptic pruning [12]. This brain imaging evidence dovetails with clinical evidence showing that several neurocognitive skills, such as working memory, divided and sustained attention, and linguistic sophistication [13], improve during this time. This suggests that the frontal lobes may be important for developing a realistic conceptualization about one's impact on the world, and the impact of the world on oneself. In addition, psychiatric disorders often first emerge during this interval of development, i.e., from adolescence to early adulthood [14]. Indeed, disorders such as bipolar and schizophrenia often have their initial onset during young adulthood [15], [16]. During this period of development, important life decisions are often made that were not directly considered at an earlier age. These decisions include, but are not limited to, determinations of whether to engage in risky behaviors such as sexual activity or substance use, or other behaviors that were previous prohibited. The concepts of the use of alcohol and other drugs are of specific interest given their cost upon society [17], [18]. Furthermore, this period of development is when substance use disorders are often initially diagnosed [19], [20]. These stages of development occur across a broad range of development from early adolescence to young adulthood (13–25). Potentially, separate aspects of neural and social development are occurring in series rather than in parallel. For example, social and emotional changes may precede changes in intellectual impact or frontal lobe development. Thus to appropriately understand the mechanisms of self/world evaluation, any scale that is developed would require inspection of the potential relationships with neural, neuropsychological, and psychological variables.

In this manuscript, we quantify adolescent/young adult participants' responses on a novel instrument, i.e., the Self/World Environment Expression Test (SWEET) that was designed to measure an individual's beliefs about self/world evaluation. First, we examine the construct validity and factor structure of the SWEET. Second, we relate responses on the SWEET to measures of psychological state (e.g., anxiety and depression), neuropsychological functioning (e.g., executive functioning and memory), and brain structure (e.g., volume and fiber integrity of the frontal lobes). We hypothesized that changes in brain structure and function that have been observed previously [7], [21] are powered not only by chronological determinants of neural development but also by brain changes related to the stress that is experienced as an adolescent shifts away from structures that reinforce a sense of personal importance. Thus, we predicted that more mature frontal lobes (i.e., smaller volume and greater white matter integrity) would relate to less of a sense of self-importance and more of a sense of the world's impact. Secondly, due to the stress involved with conceiving oneself as relatively less impactful on one's life structures, we predicted that lower ratings of the impact of the self on the world would relate to psychological measures such as more depression.

## Methods

### Participants

Sixty-five individuals (see Table 1) completed the Self/World Environment Expression Test (SWEET) and a full battery of psychological and neuropsychological measures. The group averaged 20.6 (sd±1.1; 18–23) years-old, 13.2 (sd±1.2) years of education, having more males (n = 41) than females (n = 24), and was primarily white (n = 43) non-hispanic (n = 43). A subset (n = 61) also completed high-resolution anatomical and diffusion tensor imaging brain scans. This study was completed on a subset of a larger study that inspected substance use in older adolescents; as such these samples represented both drug using and drug-abstaining individuals. These samples allowed for the inspection of the concepts of self/world in the context of substance use. To explore potential confounds of drug-users versus drug-abstainers, demographic variables were contrasted between groups. The groups differed significantly only on years of education and self-reported grade point average (see Table 1). Subjects were recruited from an on-going NIDA funded study (SFT).

**Table 1:** Demographic variables.

Variable	Non-Using Controls (n = 29)	Substance Users (n = 36)	t/chi	df	P
**Age**	20.6(1.2)	20.6(1.1)	0.047	54.8	0.963
**Education**	13.8(1.2)	13.1(1.1)	2.260	57.4	0.028
**Grade point average**	3.1(0.8)	2.9(1.0)	1.100	62.3	0.275
**Hollingshead**	26.9(13.7)	27.1(16.9)	−0.056	63.0	0.955
**Beck Depression Inventory**	2.6(4.4)	2.7(4.3)	−0.085	58.9	0.932
**State-Trait Anxiety Inventory: State**	38.3(7.5)	38.0(6.3)	−0.134	55.0	0.894
**NEO-FFI Agreeableness**	41.7(7.8)	41.0(5.3)	0.385	41.2	0.702
**NEO-FFI Conscientiousness**	43.3(6.7)	40.7(6.2)	1.538	53.7	0.130
**NEO-FFI Extroversion**	45.5(5.1)	43.8(5.4)	1.231	57.3	0.223
**NEO-FFI Neuroticism**	29.0(6.9)	29.9(7.2)	−0.456	57.2	0.650
**NEO-FFI Openness**	42.1(5.5)	43.3(5.3)	−0.835	54.9	0.407
**Gender**					
**Female**	11	13	0.023	1	0.880
**Male**	18	23			
**Race**					
**American Indian, Alaska native**	1	1	4.320	5	0.504
**Native Hawaiian, Pacific islander**	0	1			
**Black or African American**	1	0			
**White**	21	22			
**More than one race**	6	10			
**Unknown**	0	2			
**Hispanic or Latino**					
**Yes**	8	11	0.266	2	0.876
**No**	20	23			
**Unknown**	1	2			

Note. Values are provided as means (standard deviations) were appropriate.

### Ethics Statement

This study was approved by and conducted in accordance with the UCSD Human Research Protections Program.

### Measures

#### Self/World Environment Expressions Test (SWEET)

The SWEET is a 10-item visual analog scale (see File S1) that quantifies for a given individual the impact of the self on the world and the world on the self across several domains: emotional (i.e., your/everyone else's personal mood), social (i.e., your/everyone else's relationship with others), intellectual, financial, and spiritual. In addition, 2 items inquiring the impact of others both on the world and the world upon them were included in the scale as potential norming variables. World was explained to mean “the world as a whole or the global community.” These last 2 items are retained as test items for future utility but are not a core part of the questionnaire (see File S1).

#### Psychological measures

The State-Trait Anxiety Inventory (STAI; [22]) and Beck Depression Inventory (BDI-II; [23]) were used to measure anxiety and mood. The NEO-Five Factor Inventory (NEO-FFI; [24]) was used to measure personality in 5 dimensions: Extraversion, Agreeableness, Conscientiousness, Neuroticism, and Openness to Experience. These measures are well-validated and have strong psychometric properties.

#### Neuropsychological tests

To measure neuropsychological performance, the California Verbal Learning Test version II (CVLT-II: [25]: Trials 1–5, Short Free Recall, Long Free Recall) and Delis-Kaplan Executive Function System (D-KEFS; [26]: FAS Total, Stroop Inhibition, Towers Total Score, Trails 1–5 summed, Trails Switching Scaled) were administered. The CVLT is reliable and well-validated measure of verbal learning and memory. Specifically, the interviewer reads a list of 16 words to the participant and the number of correct recollections through 5 trials is scored. Short and long (∼20 minutes) free and cued recall tests are also required. The D-KEFS was used to test executive functioning. Specifically, the Verbal Fluency test (letter only) requires an individual to produce as many words beginning with a single letter as possible within a minute. The Stroop Test requires the participant to inhibit a learned behavior (i.e., reading a color name) and rather identify the word color. The Towers Test requires moving 5 disks across 3 pegs to a new predetermined arrangement in as few allowable moves as possible. Finally the Trail Making Test requires connecting dots in sequence. The switching component involves alternating the selection of sequential letters and numbers. These tests were selected to best measure frontal lobe functioning (i.e., word generation, inhibition, planning, flexibility, ability to follow rules).

#### Brain volume indices

Scans were acquired on a 3 Tesla CXK4 scanner from General Electric (Milwaukee, WI) using an eight-channel head array coil. A sagittal high resolution Spoiled Gradient Recalled anatomical sequence was acquired at the beginning of each session (25 cm field of view; 256×256 matrix; 172 1.0 mm thick slices; with 4.8 ms echo time, and 8 ms repetition time).

#### Voxel-based morphometry (VBM) analysis

Structural data was analyzed with FSL-VBM, a voxel-based morphometry style analysis [27], [28] carried out with FSL tools [Smith 2004]. First, structural images were brain-extracted using BET [29]. Next, tissue-type segmentation was carried out using FAST4 [30]. The resulting grey-matter partial volume images were then aligned to MNI152 standard space using the affine registration tool FLIRT [31], [32], followed optionally by nonlinear registration using FNIRT [33], which uses a b-spline representation of the registration warp field [34]. The resulting images were averaged to create a study-specific template, to which the native grey matter images were then non-linearly re-registered. The registered partial volume images were then modulated (to correct for local expansion or contraction) by dividing by the Jacobian of the warp field. The resultant data was resampled to 2 mm cubic voxels. The average voxel blur for all individuals was calculated using Analysis of Functional NeuroImages (AFNI) [35] function 3dFWHMx (FWHM: x = 8.43, y = 10.17, z = 9.06) and Monte Carlo (iterations = 10,000) using AlphaSim was used to determined that a cluster size of 542 voxels was required to control for multiple comparisons maintaining an alpha of .05. For contrast had tracing of frontal regions was also performed (see File S2 and Table S1).

#### White matter integrity indices

Diffusion tensor imaging (DTI) studies were acquired on a 3T General Electric scanner using an 8-channel head coil (TR = 12400 ms, TE = 99 ms, b-values = 2,000 s/mm^2^, diffusion gradient directions = 15, FOV = 24 cm, matrix = 128×128, slice thickness = 3 mm, averages = 4). The diffusion-weighted datasets were preprocessed and subjected to tensor decomposition, as in our recent studies [10]. This included corrections for head motion, eddy current distortion, and signal loss using FSL tools (FMRIB Software Library, Oxford, United Kingdom; [36]). Scalar diffusion indices, FA, MD, RD, and AD, were computed in native coordinate space using AFNI's diffusion routine, 3dDWItoDT, and were examined with Tract-Based Spatial Statistics (TBSS; [37]). TBSS analyses involved the following steps: to achieve initial alignment, FA maps were registered to an averaged FA template (FMRIB-58) in MNI-152 standard space using an affine-only registration. This was followed by a non-linear transformation into 1-mm cubic voxel dimensions (FNIRT, FMRIB's Non-linear Registration Tool). Data were examined for laterality, orientation, and cross-subject anatomical alignment. Next, transformed images were averaged across participants to create a mean diffusion image (FA), from which a white matter skeleton was derived, representing tracts common to all participants. Individual transformed FA images were then projected onto the skeleton. To minimize partial volume effects and areas of high inter-subject variability, values were thresholded at FA>0.2. FA values from individuals' nearest relevant tract center were assigned to the skeleton via a perpendicular search for the maximum FA value within the local skeleton structure. This process accounts for residual misalignments between participants after the initial registration, and minimizes systematic differences in tract location between groups of participants. MD, RD, and AD data were processed using the same non-linear transformation, skeleton, and skeleton-projection vectors derived from the FA analysis [38]. Data from each point on the skeleton formed the basis of voxel-wise statistical comparisons.

### Data Analysis

#### Psychometric properties of the SWEET

Two analyses were performed to aid in understanding the psychometric properties of the SWEET: (1) a Cronbach's alpha for the scale determined the scale reliability: and (2) a factor analysis determined appropriate subscales. The goal of these analyses was to determine the robustness of the measure and whether conceptual combinations of the items were validated by the underlying structure of the measure in an young adult sample.

Factors determined from the previous analysis were then correlated with specific psychological and neuropsychological measures as an initial inspection of the proposed constructs. From the VBM data, a multiple linear regression analysis, using AFNI program 3dRegAna, was performed with gray matter densities as a criterion variable and SWEET subscales as predictor variables. Additional correlations were performed between grey matter densities and the Self+World average and Self/(Self+World) ratio. From the DTI data, a multiple linear regression analysis was performed with fractional anisotropy (FA), an index of white matter tract coherence and integrity, as a criterion variable and SWEET subscales as predictor variables. Additional correlations were performed between white matter integrity and the Self+World average and Self/(Self+World) ratio. The interactions of all correlations with drug use were also examined. Bonferonni correction was not applied, and these evaluations were considered exploratory.

## Results

### Psychometric Properties of the SWEET

**Figure 1:**
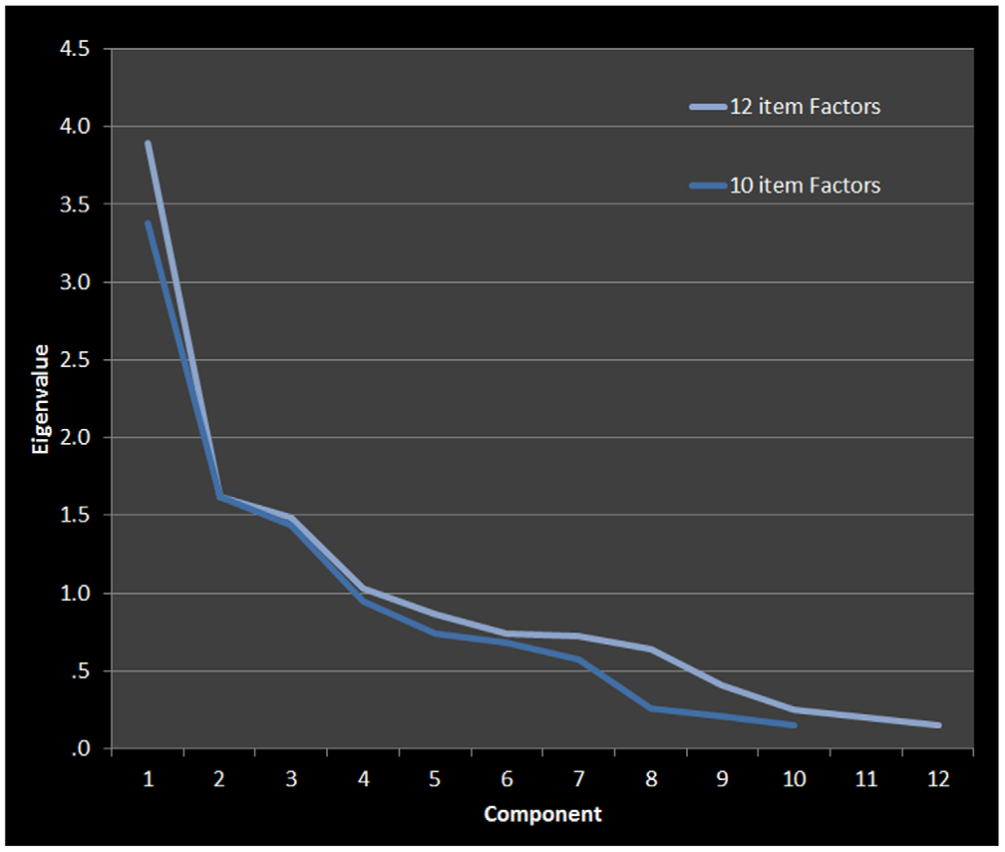
Un-rotated Factor Structure for 10 and 12 item.

All scale statistics were performed in PASW Statistics v. 17.0.3 (SPSS: An IBM Company). With all 12 items included, a Cronbach's alpha of .80 was achieved. The SWEET achieved a Cronbach's alpha of .77 using 10 items (excluding 2 items inquiring on the impact of others, as they were included as potential norming items and are not domain-specific). Cronbach's alpha with item deletion was also investigated (see Table S2). The 10 items formed a 3-factor model (using an eigenvalue cut-off of 1.0), while the 12-item model yielded a 4-factor model. Factor analysis was done is PASW, using a standard factor analytic approach [39]. On further inspection of the factors, a 4-factor model was calculated for the retained 10-item to determine the consistency of this factor model. The factor analysis (see Figure 1), varimax rotated factors (see Table 2), and inter-item correlations (see Table S3) suggest that the SWEET may comprise 4 definable constructs: (1) Self Impact, the impact of self across emotional, social, intellectual, and financial domains, (2) Social-Emotional Impact, the impact of the world on the self socially and emotionally, (3) Financial-Intellectual Impact, the impact of the world on the self financially and intellectually, and (4) Spirituality, the impact of the self on the spiritual world and vice versa. The 4 factor model was selected for three key reasons: 1) the percent variance explained by the 4 factor models was superior to the 3 factor model (74% versus 64%), 2) it provided more distinct factors and logically coherent factors, and 3) there were fewer partial correlations of items across multiple scales. Cronbach's alpha was calculated for each factor (see Table S4). While Self and Spirituality showed good alphas, the world items (i.e., Social-Emotional and Financial-Intellectual Impact) did not show a strong reliability. To look for more general trend with the scale the average SWEET score (for the ten items; Self+World) and the ratio of the total self-items to the total scale (i.e., Self/(Self+World)) were calculated for further analyses. It is important to note that alternative factor solutions were available for the 10-item SWEET. These solutions and factor structures may prove to be more informative in future studies, or may improve conceptualization of the self/world conceptualization in other samples.

**Table 2:** Varimax rotations of 4 factor models for the SWEET.

Item	Component (12-item)				Component (10-item)			
	1	2	3	4	1	2	3	4
**Emotional Impact (Your)**	**.698**	.262	−.253	.304	**.674**	.314	−.248	.323
**Emotional Impact (World)**	−.024	.184	−.115	**.822**	−.076	.229	−.122	**.806**
**Social Impact (Your)**	**.803**	.146	−.097	.139	**.824**	.120	−.077	.208
**Social Impact (World)**	.183	.078	.376	**.673**	.188	.000	.379	**.745**
**Intellectual Impact (Your)**	**.695**	.333	.270	−.169	**.749**	.219	.327	−.081
**Intellectual Impact (World)**	.055	.443	**.711**	−.018	.117	.284	**.775**	.058
**Financial Impact (Your)**	**.735**	.057	.095	−.203	**.767**	.004	.116	−.138
**Financial Impact (World)**	.017	−.057	**.825**	.064	−.024	−.065	**.799**	.041
**Spiritual Impact (Your)**	.498	**.753**	−.059	.049	.460	**.822**	−.013	.005
**Spiritual Impact (World)**	.054	**.856**	.135	.316	.033	**.869**	.208	.276
**Average Person Impact (World)**	.645	.058	.224	.307				
**World Impact on Average Person**	.439	−.155	.365	.119				
**Eigenvalue (varimax)**	3.05	1.77	1.70	1.53	2.55	1.73	1.63	1.46
**% of variance (varimax)**	25	15	14	13	26	17	16	15

Note. Bold and underlined values are assigned to the cluster column.

### SWEET scores

The SWEET was completed by 36 substance users and 29 non-using controls. The groups did not differ on the scale when controlling for multiple comparisons (see Table 3). However, there was a trend towards a difference with regard the spiritual impact of the world on the self (t(59.9) = −2.385, p = 0.020).

**Table 3:** SWEET score averages and comparison between non-using controls and substance users.

	All Participants (n = 65)		Non-Using Controls (n = 29)		Substance Users (n = 36)				
SWEET	Mean	SD	Mean	SD	Mean	SD	t	df	p
**SWEET Factors:**									
**Self Impact**	66.8	29.4	67.4	27.1	66.3	31.5	0.156	62.7	0.876
**Social-Emotional Impact**	79.4	23.9	76.6	24.2	81.7	23.7	−0.851	59.6	0.398
**Financial-Intellectual Impact**	91.9	27.2	90.8	29.8	92.9	25.4	−0.295	55.3	0.769
**Spirituality**	66.7	44.1	55.1	43.2	76.0	43.1	−1.943	60.0	0.057
**SWEET Totals:**									
**Self+World**	74.3	21.3	71.5	21.1	76.6	21.5	0.971	60.6	0.336
**Self/(Self+World)**	.42	.14	.44	.13	.41	.14	−0.785	62.5	0.436
**SWEET Items:**									
**Emotional Impact (Self)**	64.6	38.9	62.9	37.1	65.9	40.8	−0.308	62.0	0.759
**Emotional Impact (World)**	77.5	32.2	75.2	32.6	79.4	32.3	−0.516	59.9	0.607
**Social Impact (Self)**	76.4	33.9	79.6	31.4	73.9	36.1	0.673	62.6	0.504
**Social Impact (World)**	81.3	26.1	78.0	27.3	84.0	25.2	−0.913	57.8	0.365
**Intellectual Impact (Self)**	73.9	39.4	73.3	35.4	74.4	42.8	−0.110	62.9	0.912
**Intellectual Impact (World)**	97.6	30.0	93.4	28.5	101.0	31.2	−1.019	62.0	0.312
**Financial Impact (Self)**	52.1	39.1	53.7	39.6	50.8	39.1	0.303	59.8	0.763
**Financial Impact (World)**	86.3	34.9	88.2	38.8	84.8	31.8	0.386	53.9	0.701
**Spiritual Impact (Self)**	63.7	48.9	55.6	50.5	70.3	47.3	−1.205	58.3	0.233
**Spiritual Impact (World)**	69.6	47.1	54.6	45.7	81.7	45.3	−2.385	59.9	0.020
**SWEET Norming Items:**									
**Average Person Impact (World)**	63.9	39.5	64.6	39.3	63.3	40.2	0.136	60.6	0.893
**World Impact on Average Person**	107.3	27.1	105.7	27.7	108.5	27.0	−0.416	59.4	0.679

### Age and Education Correlations with the SWEET

To investigate the effects of adolescent to adult development on the SWEET, the 4 factors were correlated with age. While no factor correlated significantly with age, there was a trend for Spirituality (*r* = .21, *p* = .10). This was only mildly changed when drug (*r_p_* = .21, *p* = .09) and education (*r_p_* = .32, *p* = .009) were used as covariates.

### Psychological Correlations with the SWEET

Mood state variables STAI and BDI were correlated with the 4 factors output from the SWEET for the combined group (*N* = 65; see Table 4). While almost universally these correlated negatively, only Self Impact and Self/(Self+World) was significantly correlated with BDI (*p* = 0.016 and 0.035, respectfully). To better determine which individual items showed the greatest correlation, each SWEET item was correlated separately. In this analysis, only the social impact of self-correlated significantly (*p* = 0.002) with BDI scores. Partial correlation controlling for drug use status did not affect these relationships. Significant correlations were found for NEO-FFI subscales Extraversion with Self Impact (p = .021), Openness (p = .014) and Agreeableness (p = .011) with the Financial-Intellectual Impact, Agreeableness (p = .045) with the Self+World, and for Conscientiousness with Self Impact (p = .012) and Self/(Self+World) (p = .031). No Correlations survived a Bonferroni correction for multiple comparisons.

**Table 4:** Correlations with SWEET factor scores and psychological variables.

	SWEET Factors				SWEET Totals	
Scales	Self Impact	Social-Emotional Impact	Financial-Intellectual Impact	Spirituality	Self+World	Self/(Self+World)
**BDI**	−0.301*	−0.030	−0.105	−0.076	−0.231	−0.266*
**STAI**	−0.082	−0.083	−0.125	−0.023	−0.105	−0.048
**NEO-FFI Neuroticism**	−0.155	−0.009	−0.172	0.030	−0.119	−0.141
**NEO-FFI Extraversion**	0.292*	0.006	0.161	0.072	0.235	0.197
**NEO-FFI Openness**	0.051	0.180	0.309*	0.115	0.195	−0.115
**NEO-FFI Agreeableness**	0.250	0.143	0.325*	0.006	0.258*	0.147
**NEO-FFI Conscientiousness**	0.319*	0.099	0.116	0.014	0.235	0.276*

Note.

* = values are significant at *p*<.05.

### Neuropsychological Correlations with the SWEET

The four primary factors of the SWEET were correlated with several measures from the CVLT-II (Trials 1–5, Short Free Recall, Long Free Recall) and DKEFS (Letter Fluency Total, Stroop Inhibition, Towers Total Score, Trail Making Test Trials 1–5 total, and Trails Switching scaled scores). These resulted in no significant correlations (see Table 5); this was only mildly changed when drug and education were used as covariates.

**Table 5:** Correlations with SWEET and neuropsychological variables.

	SWEET Factors				SWEET Totals	
Scales	Self Impact	Social-Emotional Impact	Financial-Intellectual Impact	Spirituality	Self+World	Self/(Self+World)
**CVLT Trials 1–5**	−0.105	−0.005	0.142	−0.089	−0.064	−0.190
**CVLT Long Free Recall**	0.048	0.197	−0.008	0.151	−0.001	−0.160
**CVLT Short Free Recall**	−0.045	0.208	0.054	−0.081	0.134	−0.041
**FAS Total**	−0.041	−0.074	−0.196	−0.195	−0.172	−0.039
**Stroop Inhibition**	−0.092	−0.046	0.031	−0.191	−0.137	−0.070
**Towers Total**	−0.057	−0.029	0.047	−0.087	−0.065	−0.071
**Trails 1–5 summed**	0.046	−0.170	0.013	−0.106	−0.053	0.017
**Trails Switching**	−0.191	0.168	0.153	−0.047	−0.055	−0.230

Note. No values are significant at *p*<.05.

#### VBM Correlations with the SWEET

**Figure 2:**
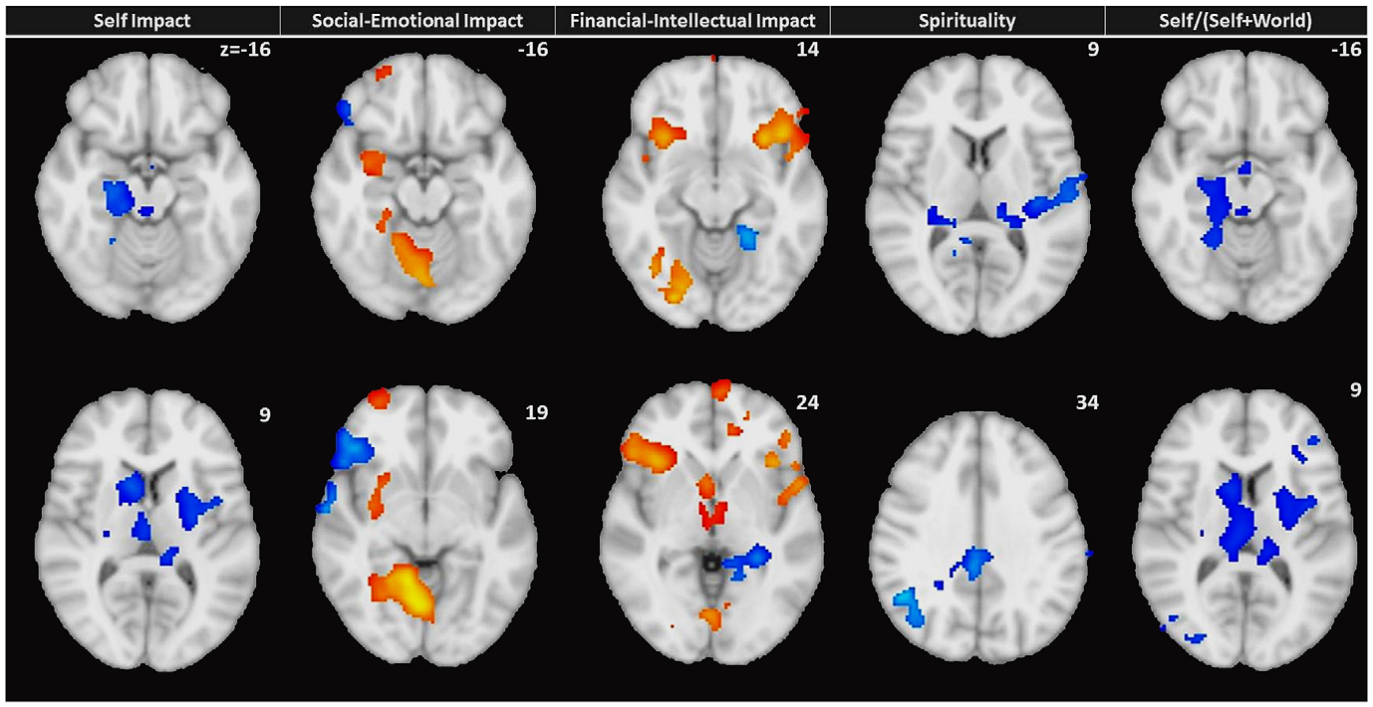
VBM: Multiple regression results for SWEET factors Self Impact [1^st^ column], Social-Emotional Impact [2^nd^ column], Financial-Intellectual [3^rd^ column], Spirituality [4^th^ column], and Self/(Self+World) [5^th^ column].

In a multiple regression with grey matter density Self Impact positively correlated with right supramarginal and inferior temporal gyrus, and negatively correlated with right occipital gyrus/culmen/amygdala, right caudate, right superior frontal gyrus, and left lentiform nucleus. Social-Emotional Impact positively correlated with right occipital gyrus/culmen/amygdala, left superior frontal gyrus, right superior frontal gyrus and negatively correlated with right inferior frontal gyrus, right middle temporal gyrus, right inferior frontal gyrus, left precuneus, left cerebellum, and left inferior parietal lobule. Financial-Intellectual Impact positively correlated with left inferior frontal gyrus, right cingulate gyrus, left cuneus, right middle temporal gyrus, right inferior frontal gyrus, left cerebellum, and right cerebellum and negatively correlated with left lingual gyrus. Spirituality correlated negatively with right cingulate gyrus, left superior temporal gyrus, and right cerebellum and did not have any significant clusters with positive correlations. No significant clusters correlated positively or negatively with Self+World. Self/(Self+World) negatively correlated with right Superior Frontal Gyrus, right Middle Occipital Gyrus, and left Middle Frontal Gyrus GM density (see Table 6; Figure 2).

**Table 6:** Multiple regression of SWEET factors predicting grey matter density.

SWEET Factors	Voxels	X	Y	Z	t-value	Region	BA
**Self Impact**	749	48	−56	30	2.427	R Supramarginal Gyrus	39
	655	54	−13	−35	2.651	R Inferior Temporal Gyrus	20
	4626	23	−40	−25	−2.455	R Occipital Gyrus/Culmen/Amygdala	19
	1010	11	8	12	−2.612	R Caudate	
	823	17	21	52	−2.460	R Superior Frontal Gyrus	6
	783	−29	−2	8	−2.396	L Lentiform Nucleus	13
**Social-Emotional Impact**	4669	13	−54	−2	2.647	R Occipital Gyrus/Culmen/Amygdala	19
	782	−21	24	52	2.528	L Superior Frontal Gyrus	8
	592	28	56	−3	2.504	R Superior Frontal Gyrus	10
	2428	51	6	25	−2.499	R Inferior Frontal Gyrus	9
	1208	51	0	−35	−2.665	R Middle Temporal Gyrus	21
	939	47	31	−6	−2.591	R Inferior Frontal Gyrus	47
	935	−8	−75	40	−2.427	L Precuneus	7
	718	−26	−36	−35	−2.353	L Cerebellum	
	554	−53	−37	26	−2.392	L Inferior Parietal Lobule	40
**Financial-Intellectual Impact**	5963	−36	23	−8	2.459	L Inferior Frontal Gyrus	47
	3926	3	0	34	2.493	R Cingulate Gyrus	24
	1540	−1	−80	12	2.687	L Cuneus	17
	1463	45	5	−26	2.702	R Middle Temporal Gyrus	21
	1356	40	26	−3	2.761	R Inferior Frontal Gyrus	47
	1204	−29	−73	−50	2.517	L Cerebellum	
	1184	22	−75	−20	2.305	R Cerebelum	
	940	−20	−47	−1	−2.391	L Lingual Gyrus	19
**Spirituality**	2557	16	−41	34	−2.520	R Cingulate Gyrus	31
	2481	−51	−21	1	−2.497	L Superior Temporal Gyrus	21
	1008	30	−28	−55	−2.239	R Cerebelum	
**Self+World**	-	-	-	-	-	-	-
**Self/Self+World**	1030	15	19	53	−2.540	R Superior Frontal Gyrus	6
	656	35	−81	11	−2.384	R Middle Occipital Gyrus	19
	614	−44	32	18	−2.287	L Middle Frontal Gyrus	46

Note. Abbreviations: L, Left; R, Right.

### DTI Correlations with the SWEET

**Figure 3:**
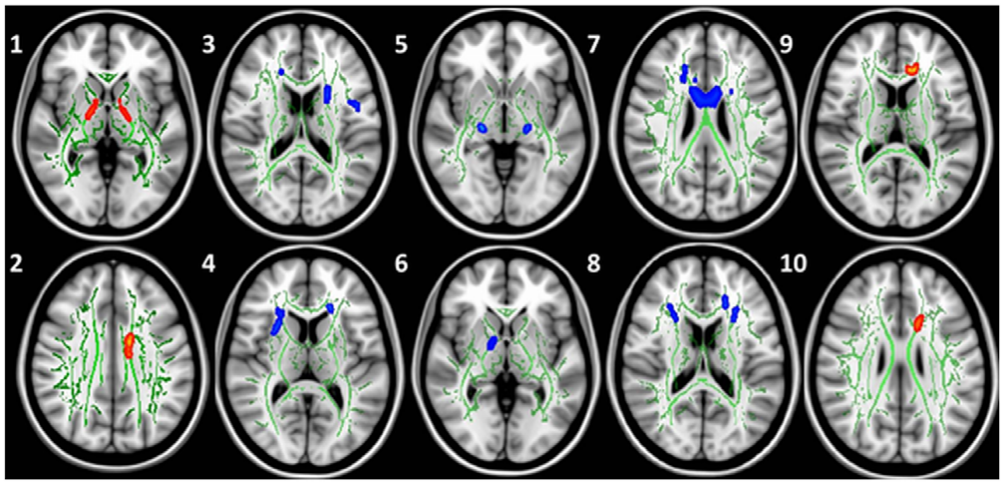
DTI: Multiple regression results for SWEET factors Self Impact [**1]**, [2], [3], [4], [5****], Social-Emotional Impact [**7]**, [8****], Financial-Intellectual [**9]**, [10****], and Spirituality [**6**].

In a multiple regression with FA, Self Impact positively correlated with bilateral anterior thalamic radiation, left superior longitudinal fasciculus, and left corpus callosum, while the left corticospinal tract, right inferior fronto-occipital fasciculus, left uncinate fasciculus, right corticospinal tract, and left forceps minor showed negative correlations. Social-Emotional Impact only showed negative correlations, primarily in the bilateral corpus callosum, as well as in the right cingulum, left corticospinal tract, left anterior thalamic radiation, right forceps minor, and left superior longitudinal fasciculus. Financial-Intellectual Impact positively correlated with greater FA in the left corpus callosum, cingulum, and forceps minor. Spirituality correlated negatively with right anterior thalamic radiation (see Table 7; Figure 3). There were no correlations with either Self+World average or Self/(Self+World) ratio.

**Table 7:** Multiple regression of SWEET factors predicting white matter integrity indices.

SWEET Factors	Voxels	X	Y	Z	t-value	Tract
**Self Impact**	421	−13	−4	−2	2.487	L Anterior Thalamic Radiation
	235	15	−4	0	2.398	R Anterior Thalamic Radiation
	222	26	25	12	−2.482	L Corticospinal Tract
	175	−1	−18	−10	−2.776	Anterior Thalamic Radiation
	146	−16	−21	−10	−2.820	L Corticospinal Tract
	146	29	11	8	−2.521	R Inferior Fronto-Occipital Fasciculus
	117	−33	−6	43	2.563	L Superior Longitudinal Fasciculus
	110	−27	23	20	−2.437	L Uncinate Fasciculus
	106	18	−22	−7	−2.569	R Corticospinal Tract
	102	−19	30	12	−2.431	Forceps Minor
	99	−13	−4	31	2.331	Corpus Callosum
**Social-Emotional Impact**	797	1	7	25	−2.649	Corpus Callosum
	136	9	7	32	−2.574	Cingulum
	131	−11	−13	−14	−2.317	L Corticospinal Tract
	111	−21	12	17	−2.533	L Anterior Thalamic Radiation
	105	19	26	23	−2.663	Forceps Minor
	90	−45	1	19	−2.371	L Superior Longitudinal Fasciculus
**Financial-Intellectual Impact**	119	−15	16	28	2.379	Corpus Callosum, Cingulum
	98	−13	30	13	2.300	Corpus Callosum, Forceps Minor
**Spirituality**	104	13	−1	2	−2.375	R Anterior Thalamic Radiation
**Self+World**	-	-	-	-	-	-
**Self/Self+World**	-	-	-	-	-	-

Note. Abbreviations: L, Left; R, Right.

## Discussion

This study makes several main points 1) the SWEET scale appears to have relatively strong internal reliability, 2) the items administered can be divide into 4 primary factors incorporating the impact of (a) self on the world, (b) the world on the self affectively (i.e., Social-Emotional), (c) the world on the self cognitively (i.e., Intellectual-Financial), and (d) the combined impact (Self+World) of Spirituality, 3) the factors of the SWEET measures do not relate strongly to drug use, psychiatric factors, or neuropsychological measures after correcting for multiple comparisons, and 4) there were some initial indications that frontal lobe volume and underlying white matter tracks related to factors of the SWEET; most notably the impact of self on the world and the affective impact of the world on self. These data suggest that the SWEET is a relatively robust measure that provides a unique piece of information that appears to have some notable neurocorrelates. Additionally, future research may want to focus on how these data relate to other psychiatric disorders such as bipolar and schizophrenia which show onset in young adulthood.

The SWEET seeks to capture how the individual evaluates their personal impact on their surroundings and the reciprocal effect upon themselves. Using a visual analog scale, we evaluated several domains where individuals interact with society at large. Through factor analysis we reduced this down to four primary factors that cover the impact of self on the world, the world's impact on self in emotional, intellectual, and spiritual concerns. This suggests that the impact of self is a generalizable, non-domain specific, construct, while the world's impact on self can be separated into affective and non-affective components. Of note, the impact of spirituality was most discretely constructed as a single factor, unlike other domains, in which the impact of the world on the self and the impact of the self on world were inversely related. To obtain a better understanding of these factors, we extended our work to inspect how these factors related to existing psychological, neuropsychological, and neural metrics.

The SWEET is not a measure of psychological well being, or a diagnostic measure of psychiatric disease. As such the SWEET does not correlate strongly with the psychiatric measures. The relationship between the BDI and lower emotional impact of self, may suggest that conceiving of oneself as less impactful on the world may contribute to a depressed mood. Inversely, conceiving of the self as being influenced by the world intellectually and financially may be an important aspect of appreciating the world thus relating to one's openness and agreeableness. While these correlations are week and conclusions they produce are speculative they may help suggest underlying aspects of the SWEET and path the way for future studies. Future studies using personality measures that measure self-concept may more effectively frame the shifting self/world concepts that occur during final stages of neural development during adolescence/young adulthood that the SWEET was designed to tap.

The items on the SWEET did not correlate with neuropsychological measures suggesting that the concept of self/world may not relate to one's intellectual or cognitive capacity. However several significant correlations were observed between the items on the SWEET and GM indices within the frontal lobes. Development from adolescence to adulthood involves systematic changes in structure and function of the frontal lobes [40], [41]. The SWEET may contribute to our understanding of the interplay between normal self/world development and neurodevelopment. Specifically, significant inverse correlations were observed in the amygdala and hippocampus with Self Impact and Self/(Self+World). This suggests that those who see large impact of self on the world have smaller amygdale. Conversely, Social-Emotional Impact of the world (i.e., the impact of the world on the self socially and emotionally) correlated with larger amygdale/hippocampal GM densities. This matches studies in adult volumetric data in depression [42] in which larger amygdala and/or hippocampal volume is found in medicated depressed subjects versus controls. Neuroanatomical studies have linked the amygdala with emotional processing and fear conditioning in particular [43]. However, the relationship between depression and Self Impact is relatively weak and findings regarding amygdala and hippocampal volume in depression have been variable. Therefore, replication of these findings is necessary to interpret these relationships in the appropriate context. Multiple regressions also demonstrated independent contributions of both the notion of self and the impact of the world to measures of GM, raising the possibility that these factors may contribute to independently, or are an effect of, underlying brain structure. These relationships illustrate the ability of the SWEET to tap into processes related to adolescent/young adult self-concept formation within expanding social structures.

Another intriguing duality exists in the relationship between Social-Emotional Impact and Financial-Intellectual Impact and the inferior frontal gyrus and anterior insula. Reductions in the grey matter of the inferior frontal gyrus in relation to greater Social-Emotional Impact may, as suggested by the related changes in amygdale/hippocampal volume, indicate contributions of these volumes to the development of psychopathology. Indeed reduced frontal volume has been related to depression [44] and acquisition of PTSD [45]. Functional imaging studies have shown that the inferior prefrontal gyrus is an important region for regulating emotions [46], social intelligence [47], and general intelligence [48], [49], [50]. While the reduced volumes in the relationship to Social-Emotional Impact may reflect a propensity for feeling overwhelmed, greater volume in relationship to Financial-Intellectual Impact may reflect that those who are more interested in intellectual pursuits may be more cognizant of the cognitive impact of others. Taken together these anatomical correlates suggest that these factors have separate neural underpinnings and thus these factors may influence how individual's conceptualize themselves within the larger environment.

Several significant correlations were also observed between the items on the SWEET and WM indices within the frontal lobes. In the DTI data, the SWEET's Self Impact subscale showed the strongest correlations with the WM (see Figure 3) suggesting that possessing the understanding that those around, and the world as a whole, have a significant impact on the self may be linked to WM development. Interestingly, as opposed to the GM findings, both the Self Impact and the Social-Emotional Impact show an inverse correlation to WM integrity (i.e., FA) in the anterior thalamic radiations. The anterior thalamic radiation is the primary path from the ventral medial frontal cortex, inferior frontal gyrus, and dorsolateral prefrontal cortex to the thalamus [51] and shows reductions in relation to psychiatric conditions such as schizophrenia and bipolar disorder [52], [53]. In previous research we have found increases in this tract during adolescence longitudinally [21], the current research suggests that failure to increase FA in this region may accompany distortions of self world concept along affective domains. The potential linkage between the SWEET and schizophrenia or drug use and brain structure is underlined in a recent imaging study [54]. James and colleagues (2011) compared control and schizophrenia adolescent subjects with and without a history of heavy cannabis use. They found widespread reduction of FA in schizophrenic subjects compared to control subjects in regions including the anterior thalamic radiation and corpus callosum; cannabis use was associated with WM reductions in these regions. Current findings provide additional evidence underscoring the relationship between measures of WM integrity and adaptive adolescent cognitive-social-emotional development.

The largest white fiber bundle that showed significant relationship to a SWEET factor was the inverse correlation between corpus callosum and the emotional impact of the world. Similar reductions in FA were associated with maltreated children with Posttraumatic Stress Disorder [55], and adults with Bipolar Depressive Disorder [56]. Interestingly, similar reduction of FA in the corpus callosum was observed in OCD [57]. Rasmussen and Eisen (1988) found that emotional environmental stressors was a common subjective precipitant of OCD [58]. In a recent study, a relationship was found between separation anxiety disorder and OCD [59], suggesting that environmental stress of changing to a larger social structure may be relevant to understanding the reduced FA in the current sample and, speculatively, the pathogenesis of OCD.

This study has several significant limitations. Primary among these limitations is the limited sample size and scope of the current study. This study takes a novel approach to understanding a scale. Rather than taking a large sample with limited data to understand the scale, a small sample of 65 is assessed across a wide range of measures and methodologies. This approach was specifically taken, as the underlying theory behind this scale was neurobiological, thus validation of the neural correlates of the scale was the key focus rather than the scale psychometrics. Future studies are planned to expand the psychometric understanding of the scale now that the anticipated neural correlates have been verified. Subsequently, the derived factors should be considered experimental at this point due to the small sample in the current study. Factors with so few items are unusual and further research may suggest that fewer factors may be more appropriate. Of specific concern are the Social-Emotional and Financial-Intellectual Impact factors, which did not show a good reliability score in the current sample. However, the interpretable meaning of these factors is bolstered by their strong neural correlates, and in light of their unique contributions to the field where this construct is not being explained by many of the existing scales and measures. The Social-Emotional and Financial-Intellectual Impact factors in particular appeared to have strong neural underpinnings in contrast with other derived factors. Based on the principles that underlie the premise of this measure it would be important to measure a large age range cross-sectionally, or preferably a single cohort longitudinally. Specifically, the sample in the current study is young adult, greater information about brain development could be garnered from a younger sample. The interpretation of the current findings should be tempered to providing insight into only the later stages of the transition between adolescents and young adulthood. However, before undertaking such an ambitious project initial evidence of the robustness of the measure and relationship to brain structure was required. We have contrasted the SWEET with common psychological measures, however it should be noted that contrasts with scales focused on assessing self-concept would greatly advance the understanding of the neuroanatomical basis of self-in-world concept. Based on this initial evidence we have begun putting into place a larger and longitudinal study on a younger sample with more psychological and neuropsychological scales for contrast.

In conclusion, the SWEET appears to be a promising measure for understanding an important and understudied aspect of an individual's experience. By understanding the perceived impact of the self and the world we can gain a greater understanding of how people respond to their environment and begin to conceptualize the individual in this context. Beyond this the SWEET may also provide some initial insight into the development of the individuals understanding of self-relevance and potentially self-worth. The initial findings relating the SWEET to brain structure is encouraging that there are neural changes that are associated with one's concept of self in context of a larger structure.

## Supporting Information

Table S1 Correlations with SWEET and frontal lobe volumes.
(DOCX)

Table S2 Cronbach's alpha if item deleted for SWEET items.
(DOCX)

Table S3 Inter item correlations with SWEET.
(DOCX)

Table S4 Cronbach's alpha for SWEET Subscales.
(DOCX)

File S1 Self/World Environment Expressions Test (SWEET).
(DOC)

File S2 Hand tracing methods and results.
(DOCX)
