# Supplementary material for: The Parametric, Psychological, Neuropsychological, and Neuroanatomical Properties of Self and World Evaluation
Source: PLoS One. 2012 Feb 13;7(2):e31509. doi: 10.1371/journal.pone.0031509 (PMC3278451; doi:10.1371/journal.pone.0031509)
Supplement: Table S1 — Correlations with SWEET and frontal lobe volumes. (DOCX) [file pone.0031509.s001.docx]

**Table S1.** Correlations with SWEET and frontal lobe volumes.

| **Analysis** | **SWEET Factors** | **Posterior dorsal PFC** | **Posterior ventral PFC** | **Anterior dorsal PFC** | **Anterior ventral PFC** |
| --- | --- | --- | --- | --- | --- |
| **Partial** | **Self Impact** | -.099 | -.067 | -.034 | -.323 |
|  | **Social-Emotional Impact** | -.065 | -.045 | -.265 | -.236 |
|  | **Financial-Intellectual Impact** | -.145 | -.334* | .009 | -.025 |
|  | **Spirituality** | -.174 | -.148 | -.153 | -.347* |
| **Non-Using Controls (*n*=29)** | **Self Impact** | -.458* | -.400 | .255 | -.276 |
|  | **Social-Emotional Impact** | -.213 | -.103 | -.341 | -.516* |
|  | **Financial-Intellectual Impact** | -.195 | -.581* | .070 | -.255 |
|  | **Spirituality** | -.356 | -.402 | -.012 | -.217 |
| **Substance Users (*n*=36)** | **Self Impact** | .391 | .345 | -.419 | -.387 |
|  | **Social-Emotional Impact** | .072 | .004 | -.199 | -.001 |
|  | **Financial-Intellectual Impact** | -.069 | .012 | -.087 | .310 |
|  | **Spirituality** | .009 | .085 | -.293 | -.464 |

Note. All volumes are provided as ROI volume over total intracranial volume. **p*<.05.
