# Supplementary material for: The Parametric, Psychological, Neuropsychological, and Neuroanatomical Properties of Self and World Evaluation
Source: PLoS One. 2012 Feb 13;7(2):e31509. doi: 10.1371/journal.pone.0031509 (PMC3278451; doi:10.1371/journal.pone.0031509)
Supplement: Table S2 — Cronbach's alpha if item deleted for SWEET items. (DOCX) [file pone.0031509.s002.docx]

**Table S2.** Cronbach's alpha if item deleted for SWEET items.

| Item | Alpha (10-item) | Alpha (12-item) |
| --- | --- | --- |
| Emotional Impact (Your) | .726 | 0.770 |
| Emotional Impact (World) | .769 | 0.801 |
| Social Impact (Your) | .727 | 0.769 |
| Social Impact (World) | .750 | 0.786 |
| Intellectual Impact (Your) | .719 | 0.764 |
| Intellectual Impact (World) | .750 | 0.788 |
| Financial Impact (Your) | .749 | 0.784 |
| Financial Impact (World) | .781 | 0.806 |
| Spiritual Impact (Your) | .704 | 0.758 |
| Spiritual Impact (World) | .728 | 0.777 |
| Average Person Impact (World) | - | 0.768 |
| Worlds Impact on Average Person | - | 0.792 |
