# Supplementary material for: The Parametric, Psychological, Neuropsychological, and Neuroanatomical Properties of Self and World Evaluation
Source: PLoS One. 2012 Feb 13;7(2):e31509. doi: 10.1371/journal.pone.0031509 (PMC3278451; doi:10.1371/journal.pone.0031509)
Supplement: Table S3 — Inter item correlations with SWEET. (DOCX) [file pone.0031509.s003.docx]

**Table S3.** Inter item **c**orrelations with SWEET.

| Item | Emotional Impact (Your) | Emotional Impact (World) | Social Impact (Your) | Social Impact (World) | Intellectual Impact (Your) | Intellectual Impact (World) | Financial Impact (Your) | Financial Impact (World) | Spiritual Impact (Your) | Spiritual Impact (World) | Average Person Impact (World) | Worlds Impact on Average Person |
| --- | --- | --- | --- | --- | --- | --- | --- | --- | --- | --- | --- | --- |
| Emotional Impact (Your) | 1 |  |  |  |  |  |  |  |  |  |  |  |
| Emotional Impact (World) | .285 | 1 |  |  |  |  |  |  |  |  |  |  |
| Social Impact (Your) | .690‡ | .039 | 1 |  |  |  |  |  |  |  |  |  |
| Social Impact (World) | .149 | .332† | .260* | 1 |  |  |  |  |  |  |  |  |
| Intellectual Impact (Your) | .401‡ | .029 | .494‡ | .153 | 1 |  |  |  |  |  |  |  |
| Intellectual Impact (World) | .075 | .046 | .104 | .278* | .455‡ | 1 |  |  |  |  |  |  |
| Financial Impact (Your) | .281* | -.099 | .406‡ | .155 | .582‡ | .045 | 1 |  |  |  |  |  |
| Financial Impact (World) | -.113 | -.048 | -.017 | .206 | .102 | .404‡ | .050 | 1 |  |  |  |  |
| Spiritual Impact (Your) | .542‡ | .138 | .478‡ | .125 | .448‡ | .188 | .355† | -.009 | 1 |  |  |  |
| Spiritual Impact (World) | .272* | .319† | .187 | .358† | .202 | .322† | .095 | .106 | .695‡ | 1 |  |  |
| Average Person Impact (World) | .418‡ | .198 | .453‡ | .298* | .449‡ | .154 | .388† | .246 | .382† | .241 | 1 |  |
| Worlds Impact on Average Person | .211 | .018 | .229 | .176 | .248 | .165 | .215 | .209 | .214 | .072 | .160 | 1 |

Note. ‡<.001; †<.01; *<.05; .25 .32 .39
