# Supplementary material for: The Parametric, Psychological, Neuropsychological, and Neuroanatomical Properties of Self and World Evaluation
Source: PLoS One. 2012 Feb 13;7(2):e31509. doi: 10.1371/journal.pone.0031509 (PMC3278451; doi:10.1371/journal.pone.0031509)
Supplement: Table S4 — Cronbach's alpha for SWEET Subscales. (DOCX) [file pone.0031509.s004.docx]

**Table S4.** Cronbach's alpha for SWEET Subscales.

| Item | Alpha |
| --- | --- |
| Self Impact | 0.780 |
| Social-Emotional Impact | 0.491 |
| Financial-Intellectual Impact | 0.575 |
| Spirituality | 0.820 |
