# Supplementary material for: The Parametric, Psychological, Neuropsychological, and Neuroanatomical Properties of Self and World Evaluation
Source: PLoS One. 2012 Feb 13;7(2):e31509. doi: 10.1371/journal.pone.0031509 (PMC3278451; doi:10.1371/journal.pone.0031509)
Supplement: File S1 — Self/World Environment Expressions Test (SWEET). (DOC) [file pone.0031509.s005.doc]

**Self/World Environment Expressions Test**

Name:_____________ Date:______________

Age:___________ Ethnicity:______________

Please decide the maximum impact you feel you have on the world, and the maximum impact that the world has on you, in each of the following areas, and then make a line (i.e., **|**) between “none at all” and “extremely.” NOTE: In this world means **the world as a whole or the global community**.

**EMOTIONAL IMPACT** (i.e., your/everyone else’s personal mood)

*What is your* ***emotional*** *impact on the world*:

| None at all |  | Moderate |  | Extreme |
| --- | --- | --- | --- | --- |
|  |  |  |  |  |
|  |  |  |  |  |

*What is the world’s* ***emotional*** *impact on you:*

| None at all |  | Moderate |  | Extreme |
| --- | --- | --- | --- | --- |
|  |  |  |  |  |
|  |  |  |  |  |

**SOCIAL IMPACT** (i.e., your/everyone else’s relationship with others)

*What is your* ***social*** impact on the world:

| None at all |  | Moderate |  | Extreme |
| --- | --- | --- | --- | --- |
|  |  |  |  |  |
|  |  |  |  |  |

*What is the world’s* ***social*** impact on you:

| None at all |  | Moderate |  | Extreme |
| --- | --- | --- | --- | --- |
|  |  |  |  |  |
|  |  |  |  |  |

**INTELLECTUAL IMPACT**

*What is your* ***intellectual*** impact on the world:

| None at all |  | Moderate |  | Extreme |
| --- | --- | --- | --- | --- |
|  |  |  |  |  |
|  |  |  |  |  |

*What is the world’s* ***intellectual*** impact on you:

| None at all |  | Moderate |  | Extreme |
| --- | --- | --- | --- | --- |
|  |  |  |  |  |
|  |  |  |  |  |

**FINANCIAL IMPACT**

*What is your* ***financial*** impact on the world:

| None at all |  | Moderate |  | Extreme |
| --- | --- | --- | --- | --- |
|  |  |  |  |  |
|  |  |  |  |  |

*What is the world’s* ***financial*** *impact on you:*

| None at all |  | Moderate |  | Extreme |
| --- | --- | --- | --- | --- |
|  |  |  |  |  |
|  |  |  |  |  |

**SPIRITUAL IMPACT**

*What is your* ***spiritual*** *impact on the spiritual* world:

| None at all |  | Moderate |  | Extreme |
| --- | --- | --- | --- | --- |
|  |  |  |  |  |
|  |  |  |  |  |

*What is the spiritual world’s* ***spiritual*** impact on you:

| None at all |  | Moderate |  | Extreme |
| --- | --- | --- | --- | --- |
|  |  |  |  |  |
|  |  |  |  |  |

**IMPACT OF OTHERS**

*What is the* ***average person’s*** impact on the world:

| None at all |  | Moderate |  | Extreme |
| --- | --- | --- | --- | --- |
|  |  |  |  |  |
|  |  |  |  |  |

*What is the world’s impact on the* ***average person***:

| None at all |  | Moderate |  | Extreme |
| --- | --- | --- | --- | --- |
|  |  |  |  |  |
|  |  |  |  |  |
