# Supplementary material for: The Parametric, Psychological, Neuropsychological, and Neuroanatomical Properties of Self and World Evaluation
Source: PLoS One. 2012 Feb 13;7(2):e31509. doi: 10.1371/journal.pone.0031509 (PMC3278451; doi:10.1371/journal.pone.0031509)
Supplement: File S2 — Hand tracing methods and results. (DOCX) [file pone.0031509.s006.docx]

Supplementary information

Brain volume indices. For a subset of 30 individuals the frontal lobes was hand delineated for further analysis. Each participant’s high-resolution anatomical image was AC-PC aligned and skull-stripped using a combination of a hybrid watershed and deformable surface semi-automated skull-stripping program [[1](#_ENREF_1)] and manual editing. Intracranial volume (ICV) was determined from the skull-stripped brain. PFC regions of interest (ROIs) were defined manually in AFNI [[2](#_ENREF_2)] by raters blind to participant characteristics who attained high levels of inter-rater reliability (intraclass correlation coefficients >.90). All ROIs were manually delineated on coronal 3D images (perpendicular to the AC-PC plane). The PFC ROIs were defined based on prior work [[3](#_ENREF_3),[4](#_ENREF_4)]. The posterior PFC ROI included all cortical areas anterior to the anterior commissure and posterior to the anterior edge of the genu. This ROI excluded the corpus callosum, subcortical regions (caudate, putamen, globus palladus, internal capsule), optic tracts, insula, and lateral ventricles. The anterior PFC ROIs (dorsal and ventral) included all cortical areas anterior to the anterior edge of the genu. The anterior dorsal PFC ROI included all cortex superior to the midline of the most anterior portion of the genu, while the anterior ventral PFC ROI included cortex inferior to the midline of the genu. Tracing continued until the most anterior slice on which cortex was still visible. These ROIs excluded the lateral ventricles and the corpus callosum. The corpus callosum was excluded from the mid-sagittal slice and then from each left and right lateral slice until the edge could no longer be detected (using AFNI’s *edge detection* and *sharpen* features).

White matter was segmented from gray and CSF compartments by processing skull-stripped T1 images with the Oxford Centre for Functional Magnetic Resonance Imaging of the Brain's FAST automated segmentation tool [[5](#_ENREF_5)]. This process uses a hidden Markov random field model and an associated expectation-maximization algorithm that corrects for spatial intensity variations. The segmentation process did not reliably separate gray matter from CSF, but reliably identified white matter. Each PFC ROI mask was applied to the white matter compartment to calculate the white matter volume for each region. All total volume and white matter ROIs were analyzed as a ratio to overall ICV to control for individual variability in brain size [[6](#_ENREF_6)].

Frontal Lobe Volume Correlations with the SWEET. Correlations were performed with SWEET factors and frontal lobe ROI volumes (see Table 6). When group was collapsed, the anterior ventral PFC volume correlated significantly with Spirituality (*r* = -.35, *p*<.05) and posterior ventral PFC volume correlated significantly with Financial-Intellectual Impact (*r* = -.33, *p*<.05). Among abstinent controls, posterior ventral PFC volume correlated with Financial-Intellectual Impact (*r* = -.52, *p*<.05), anterior ventral PFC volume with Social-Emotional Impact (*r* = -.58, *p*<.01), and posterior dorsal PFC volume with Self Impact (*r* = -.46, *p*<.05). There were no significant correlations between frontal lobe volumes and SWEET factors for substance users, and all correlations found in the control group were significantly different between substance users and controls.

Specifically, significant correlations with the frontal lobe suggest the decreased posterior ventral PFC relates to increased impact of the cognitive world. This relationship did not exist in the drug users, however the correlations only approached to be significantly different between the groups (p=0.069). Similarly, decreases in the posterior dorsal PFC positively related to increased emotional impact of self in abstainers while inversely related in users and the groups were significantly different in this relationship (p= 0.0147). Interestingly numerous mental disorders, such as Obsessive Compulsive Disorder (OCD) [[7](#_ENREF_7)], Post-Traumatic Stress Disorder (PTSD) [[8](#_ENREF_8)], and Schizophrenia [[9](#_ENREF_9),[10](#_ENREF_10),[11](#_ENREF_11)], show reduction in the posterior PFC in comparison to healthy controls.

References

1. Segonne F, Dale AM, Busa E, Glessner M, Salat D, et al. (2004) A hybrid approach to the skull stripping problem in MRI. Neuroimage 22: 1060-1075.

2. Cox RW (1996) AFNI: software for analysis and visualization of functional magnetic resonance neuroimages. ComputBiomedRes 29: 162-173.

3. Nagel BJ, Medina KL, Yoshii J, Schweinsburg AD, Moadab I, et al. (2006) Age-related changes in prefrontal white matter volume across adolescence. Neuroreport 17: 1427-1431.

4. Medina KL, McQueeny T, Nagel BJ, Hanson KL, Schweinsburg AD, et al. (2008) Prefrontal cortex volumes in adolescents with alcohol use disorders: unique gender effects. Alcoholism: Clinical and Experimental Research 32: 386-394.

5. Zhang Y, Brady M, Smith S (2001) Segmentation of brain MR images through a hidden Markov random field model and the expectation maximization algorithm. IEEE Transactions on Medical Imaging 20: 45-57.

6. Giedd JN, Snell JW, Lange N, Rajapakse JC, Casey BJ, et al. (1996) Quantitative magnetic resonance imaging of human brain development: ages 4-18. Cereb Cortex 6: 551-560.

7. Togao O, Yoshiura T, Nakao T, Nabeyama M, Sanematsu H, et al. (2010) Regional gray and white matter volume abnormalities in obsessive-compulsive disorder: a voxel-based morphometry study. Psychiatry Res 184: 29-37.

8. Quirk GJ, Garcia R, Gonzalez-Lima F (2006) Prefrontal mechanisms in extinction of conditioned fear. Biol Psychiatry 60: 337-343.

9. Egan MF, Goldberg TE, Kolachana BS, Callicott JH, Mazzanti CM, et al. (2001) Effect of COMT Val(108/158) Met genotype on frontal lobe function and risk for schizophrenia. Proceedings of the National Academy of Sciences of the United States of America 98: 6917-6922.

10. Weinberger DR, Egan MF, Bertolino A, Callicott JH, Mattay VS, et al. (2001) Prefrontal neurons and the genetics of schizophrenia. Biological Psychiatry 50: 825-844.

11. Thompson PM, Vidal C, Giedd JN, Gochman P, Blumenthal J, et al. (2001) Mapping adolescent brain change reveals dynamic wave of accelerated gray matter loss in very early-onset schizophrenia. Proceedings of the National Academy of Sciences of the United States of America 98: 11650-11655.

**Self/World Environment Expressions Test**

Name:_____________ Date:______________

Age:___________ Ethnicity:______________

Please decide the maximum impact you feel you have on the world, and the maximum impact that the world has on you, in each of the following areas, and then make a line (i.e., **|**) between “none at all” and “extremely.” NOTE: In this world means **the world as a whole or the global community**.

**EMOTIONAL IMPACT** (i.e., your/everyone else’s personal mood)

*What is your* ***emotional*** *impact on the world:*

| None at all |  | Moderate |  | Extreme |
| --- | --- | --- | --- | --- |
|  |  |  |  |  |
|  |  |  |  |  |

*What is the world’s* ***emotional*** *impact on you:*

| None at all |  | Moderate |  | Extreme |
| --- | --- | --- | --- | --- |
|  |  |  |  |  |
|  |  |  |  |  |

**SOCIAL IMPACT** (i.e., your/everyone else’s relationship with others)

*What is your* ***social*** *impact on the world:*

| None at all |  | Moderate |  | Extreme |
| --- | --- | --- | --- | --- |
|  |  |  |  |  |
|  |  |  |  |  |

*What is the world’s* ***social*** *impact on you:*

| None at all |  | Moderate |  | Extreme |
| --- | --- | --- | --- | --- |
|  |  |  |  |  |
|  |  |  |  |  |

**INTELLECTUAL IMPACT**

*What is your* ***intellectual*** *impact on the world:*

| None at all |  | Moderate |  | Extreme |
| --- | --- | --- | --- | --- |
|  |  |  |  |  |
|  |  |  |  |  |

*What is the world’s* ***intellectual*** *impact on you:*

| None at all |  | Moderate |  | Extreme |
| --- | --- | --- | --- | --- |
|  |  |  |  |  |
|  |  |  |  |  |

**FINANCIAL IMPACT**

*What is your* ***financial*** *impact on the world:*

| None at all |  | Moderate |  | Extreme |
| --- | --- | --- | --- | --- |
|  |  |  |  |  |
|  |  |  |  |  |

*What is the world’s* ***financial*** *impact on you:*

| None at all |  | Moderate |  | Extreme |
| --- | --- | --- | --- | --- |
|  |  |  |  |  |
|  |  |  |  |  |

**SPIRITUAL IMPACT**

*What is your* ***spiritual*** *impact on the spiritual world:*

| None at all |  | Moderate |  | Extreme |
| --- | --- | --- | --- | --- |
|  |  |  |  |  |
|  |  |  |  |  |

*What is the spiritual world’s* ***spiritual*** *impact on you:*

| None at all |  | Moderate |  | Extreme |
| --- | --- | --- | --- | --- |
|  |  |  |  |  |
|  |  |  |  |  |

**IMPACT OF OTHERS**

*What is the* ***average person’s*** *impact on the world:*

| None at all |  | Moderate |  | Extreme |
| --- | --- | --- | --- | --- |
|  |  |  |  |  |
|  |  |  |  |  |

*What is the world’s impact on the* ***average person****:*

| None at all |  | Moderate |  | Extreme |
| --- | --- | --- | --- | --- |
|  |  |  |  |  |
|  |  |  |  |  |
